# Supplementary material for: Oxidative stress induces stem cell proliferation via TRPA1/RyR-mediated Ca2+ signaling in the Drosophila midgut
Source: eLife. 2017 May 31;6:e22441. doi: 10.7554/eLife.22441 (PMC5451214; doi:10.7554/eLife.22441)
Supplement: Supplementary file 1. — DOI: http://dx.doi.org/10.7554/eLife.22441.037 [file elife-22441-supp1.docx]

**Supplementary File**

**List of genotypes used in each figure**

**Figure 1**

*EGT; UAS-Luc RNAi*

*EGT; UAS- trpA1* *RNAi (BL31504, in all Figures unless noted) **

*EGT; UAS-RyR RNAi*

*EGT; UAS-SERCA RNAi (BL44581, in all Figures unless noted) **/ Luc RNAi*

*EGT; UAS-SERCA RNAi **/ trpA1 RNAi*

*EGT; UAS-SERCA RNAi/ RyR RNAi*

*EGT; UAS-Cas9.P2/ attP2*

*EGT; UAS-Cas9.P2/ U6-sgtrpA1*

*EGT; UAS-Cas9.P2/ U6-sgRyR*

*Both BL31504 and v37249 lines of *trpA1* RNAi were tested and gave similar results.

**Both BL44581 and BL25928 lines of *SERCA* RNAi were tested and gave similar results.

**Figure 1—figure supplement 1**

*tubGal80^ts^; DaGal4/ UAS-Luc RNAi*

*tubGal80^ts^; DaGal4/ UAS-trpA1 RNAi (BL31504)*

*tubGal80^ts^; DaGal4/ UAS-trpA1 RNAi (BL31384)*

*tubGal80^ts^; DaGal4/ UAS-trpA1 RNAi (BL36780)*

*tubGal80^ts^; DaGal4/ UAS-RyR RNAi (BL29445)*

*tubGal80^ts^; DaGal4/ UAS-RyR RNAi (BL31540)*

*tubGal80^ts^; DaGal4/ UAS-RyR RNAi (BL31695)*

*tubGal80^ts^; DaGal4/ attP (v60100)*

*tubGal80^ts^; DaGal4/ UAS-trpA1 RNAi (v37249)*

*EGT; UAS-Luc RNAi*

*EGT; UAS- trpA1* *RNAi*

*EGT; UAS-RyR RNAi*

**Figure 1—figure supplement 2**

*EGT; UAS-Luc RNAi*

*EGT; UAS- trpA1* *RNAi*

*EGT; UAS-RyR RNAi*

*EGT; UAS-Luc RNAi*

*EGT; UAS-trpA1 RNAi*

*EGT; UAS-RyR RNAi*

*EGT; UAS-Luc RNAi/ UAS-p35*

*EGT; UAS-trpA1 RNAi/ UAS-p35*

*EGT; UAS-RyR RNAi/ UAS-p35*

**Figure 1—figure supplement 3**

*UAS-rpr/+; EGT/+*

**Figure 2**

*hsFlp tubGal80 FRT19A/ FRT19A; tubGal4 UAS-mCD8::GFP/ UAS-Luc RNAi*

*hsFlp tubGal80 FRT19A/ FRT19A; tubGal4 UAS-mCD8::GFP/ UAS-trpA1 RNAi*

*hsFlp tubGal4 UAS-GFP-myc-nls;; tubGal80 FRT2A/ FRT2A*

*hsFlp tubGal4 UAS-GFP-myc-nls;; tubGal80 FRT2A/ trpA1^1^ FRT2A*

*yw hsFlp UAS-GFP tubGal4; FRT42D tubGal80/ FRT42D* (BL8216) *

*yw hsFlp UAS-GFP tubGal4; FRT42D tubGal80/ FRT42D RyR^k04913^*

*EGT; Dl-lacZ/ UAS-Luc RNAi*

*EGT; Dl-lacZ/ UAS-trpA1 RNAi ***

*EGT; Dl-lacZ/ UAS-RyR RNAi*

*tubGal80^ts^; esgGal4 Su(H)GbeGFP; UAS-Luc RNAi*

*tubGal80^ts^; esgGal4 Su(H)GbeGFP; UAS-trpA1 RNAi ***

*tubGal80^ts^; esgGal4 Su(H)GbeGFP; UAS-RyR RNAi*

*EGT; UAS-Luc RNAi*

*EGT; UAS-trpA1 RNAi*

*EGT; UAS-RyR RNAi*

*Consistent results when two additional *FRT42D* lines, BL1928 and BL1802, are used as controls

**Both BL31504 and v37249 lines of *trpA1* RNAi were tested and gave similar results.

**Figure 2—figure supplement 1**

*hsFlp tubGal80 FRT19A/ FRT19A; tubGal4 UAS-mCD8::GFP/ UAS-Luc RNAi*

*hsFlp tubGal80 FRT19A/ FRT19A; tubGal4 UAS-mCD8::GFP/ UAS-trpA1 RNAi*

*yw hsFlp UAS-GFP tubGal4; FRT42D tubGal80/ FRT42D* (BL8216) *

*yw hsFlp UAS-GFP tubGal4; FRT42D tubGal80/ FRT42D RyR^k04913^*

*EGT; UAS-GFP, Act>>Gal/ UAS-Luc RNAi*

*EGT; UAS-GFP, Act>>Gal/ UAS-trpA1 RNAi*

*Consistent results when two additional *FRT42D* lines, BL1928 and BL1802, are used as controls

**Figure 3**

*EGT; UAS-Luc RNAi*

*EGT; UAS-trpA1 RNAi*

*Esg-lacZ; trpA1Gal4^CP2A^; UAS-mCherry*

*esgGFP UAS-mCherryCAAX; RyRGal4^R14G09^*

*esgGal4/ UAS-GCamP6f; trpA1^1^/ +*

*esgGal4/ UAS-GCamP6f; trpA1^1^/ Df4415*

**Figure 3—figure supplement 2**

*w/+; EGT/+*

*UAS-rpr/+; EGT/+*

*tubGal80^ts^; DlGal4/ UAS-Luc RNAi*

*tubGal80^ts^; DlGal4/ UAS-trpA1* *RNAi*

*tubGal80^ts^; Su(H)Gal4; UAS-Luc RNAi*

*tubGal80^ts^; Su(H)Gal4; UAS-trpA1* *RNAi*

*tubGal80^ts^; 24BGal4/ UAS-Luc RNAi*

*tubGal80^ts^; 24Bal4/ UAS- trpA1* *RNAi*

*tubGal80^ts^; tubGal4/ UAS-Luc RNAi*

*tubGal80^ts^; tubGal4/ UAS-trpA1 RNAi*

**Figure 4**

*EGT; UAS-trpA1 RNAi/ UAS-nlsGFP*

*EGT/ UAS-trpA1-C; UAS-trpA1 RNAi/ UAS-trpA1-C*

*EGT/ UAS-trpA1-D; UAS-trpA1 RNAi/ UAS-trpA1-D*

*EGT/ UAS-2xEGFP [m5B29]; UAS-trpA1 RNAi/ UAS-2xEGFP [m6B1]*

*EGT; UAS-CncC RNAi*

*EGT; UAS-CncC RNAi/ UAS-trpA1 RNAi*

*EGT;*

*EGT/ UAS-trpA1-C; UAS-trpA1-C*

*EGT/ UAS-trpA1-D; UAS-trpA1-D*

*EGT; UAS-trpA1-A*

**Figure 4—figure supplement 1**

*EGT*

*EGT; UAS-Hep^ca^*

*w; esgGFP MyO1AGal4; tubGal80^ts^*

*w; esgGFP MyO1AGal4; tubGal80^ts^/ UAS-Hep^ca^*

*UAS-rpr; esgGFP MyO1AGal4; tubGal80^ts^*

**Figure 5 & Figure 5—figure supplement 1**

*esgGal4; UAS-GCaMP6s*

*esgGal4; UAS-GCaMP6s/ UAS-trpA1 RNAi*

*esgGal4; UAS-GCaMP6s/ UAS-RyR RNAi*

**Figure 5—figure supplement 2**

*esgGal4; UAS-tdTomato-P2A-GCaMP5G*

*esgGal4; UAS-tdTomato-P2A-GCaMP5G/ UAS-trpA1 RNAi*

*UAS-RFP LexAop2-GFP; esgGal4/ UAS-MKII::nlsLexA^DBD^, UAS-p65AD::CaM; UAS-p65AD::CaM, tubGal80^ts^*

*UAS-RFP LexAop2-GFP; esgGal4/ UAS-MKII::nlsLexA^DBD^, UAS-p65AD::CaM; UAS-p65AD::CaM, tubGal80^ts^/ UAS-trpA1 RNAi*

*UAS-RFP LexAop2-GFP; esgGal4/ UAS-MKII::nlsLexA^DBD^, UAS-p65AD::CaM; UAS-p65AD::CaM, tubGal80^ts^/ UAS-RyR RNAi*

**Figure 6 & Figure 6—figure supplement 1**

*EGT; UAS-Luc RNAi*

*EGT; UAS-SERCA RNAi **

*EGT; UAS-trpA1 RNAi*

*EGT; UAS-RyR RNAi*

*EGT; UAS-SERCA RNAi/ UAS-Ras1 RNAi (BL29319) ***

*EGT; UAS-SERCA RNAi/ UAS-Yki RNAi (BL31965) ****

*EGT; UAS-SERCA RNAi/ UAS-trpA1 RNAi*

*EGT; UAS-SERCA RNAi/ UAS-RyR RNAi*

*UAS-RFP LexAop2-GFP; esgGal4/ UAS-MKII::nlsLexA^DBD^, UAS-p65AD::CaM; UAS-p65AD::CaM, tubGal80^ts^*

*UAS-RFP LexAop2-GFP; esgGal4/ UAS-MKII::nlsLexA^DBD^, UAS-p65AD::CaM; UAS-p65AD::CaM, tubGal80^ts^/ UAS-trpA1 RNAi*

*Both BL44581 and BL25928 lines of *SERCA* RNAi were tested and gave similar results.

**Both BL29319 and v106642 lines of *Ras1* RNAi were tested and gave similar results.

*** Both BL31965 and v104523 lines of *Yki* RNAi were tested and gave similar results.

**Figure 6—figure supplement 2**

*EGT*

*EGT; UAS-Src42A^ca^*

*EGT/ UAS-Src64B*

*EGT; UAS-Ras1^A^*

*EGT; UAS-trpA1 RNAi*

*EGT; UAS-Src42A^ca^/ UAS-trpA1 RNAi*

*EGT/ UAS-Src64B; UAS-trpA1 RNAi*

*EGT; UAS-trpA1 RNAi/ UAS-nlsGFP*

*EGT; UAS-SERCA RNAi (BL25928)/ UAS-Luc RNAi*

*EGT; UAS-SERCA RNAi (BL25928)/ UAS-Src42A RNAi*

*EGT; UAS-SERCA RNAi (BL25928)/ UAS-Src64B RNAi*

**Figure 7**

*EGT; UAS-nlsGFP*

*EGT; UAS-trpA1 RNAi/ UAS-nlsGFP*

*EGT; UAS-Ras1^A^*

*EGT; UAS-trpA1 RNAi/ UAS-Ras1^A^*

*EGT; UAS-CanA1^ca^*

*EGT; UAS-trpA1 RNAi/ UAS-CanA1^ca^*

*EGT; UAS-CRTC*

*EGT; UAS-trpA1 RNAi/ UAS-CRTC*

*EGT; UAS-CrebB^act^*

*EGT; UAS-trpA1 RNAi/ UAS-CrebB^act^*

*EGT; UAS-Raf^gof^*

*EGT; UAS-trpA1 RNAi/ UAS-Raf^gof^*

*EGT; UAS-spi*

*EGT; UAS-trpA1 RNAi/ UAS-spi*

*EGT; UAS-pvf1*

*EGT; UAS-trpA1 RNAi/ UAS-pvf1*

**Figure 7—figure supplement 1A-F**

*EGT; UAS-SERCA RNAi/ UAS-Luc RNAi*

*EGT; UAS-SERCA RNAi/ UAS-EGFR RNAi (BL25781)*

*EGT; UAS-SERCA RNAi/ UAS-EGFR RNAi^2^ (BL31525)*

*EGT; UAS-SERCA RNAi/ UAS-EGFR RNAi^3^ (BL31526)*

*EGT; UAS-SERCA RNAi/ UAS-Ras1 RNAi (v106642) **

*EGT; UAS-SERCA RNAi/ UAS-Yki RNAi (BL31965)*

*EGT; UAS-SERCA RNAi/ UAS-CanA1 RNAi (BL25850)*

*EGT; UAS-SERCA RNAi/ UAS-CrebB RNAi (BL63681)*

*EGT; UAS-SERCA RNAi/ UAS-CrebB RNAi^2^ (BL29332)*

*EGT; UAS-SERCA RNAi/ UAS-CRTC RNAi (BL28886)*

*Both v106642 and BL29319 lines of *Ras1* RNAi were tested and gave similar results.

**Figure 7—figure supplement 1G**

*EGT; UAS-SERCA RNAi^2^ (BL25928)/ attp (v60100)*

*EGT; UAS-SERCA RNAi^2^ (BL25928)/ UAS-CanA1 RNAi (fb5)*

*EGT; UAS-SERCA RNAi^2^ (BL25928)/ UAS-Yki RNAi (*v104523*)*

**Figure 7—figure supplement 1H-I**

*tubGal80^ts^; DaGal4/ UAS-Luc RNAi*

*tubGal80^ts^; DaGal4/ UAS-CRTC RNAi*

*tubGal80^ts^; DaGal4/ attp (v60100)*

*tubGal80^ts^; DaGal4/ UAS-CanA1 RNAi (fb5)*

**Figure 7—figure supplement 2**

*EGT; UAS-Luc RNAi*

*EGT; UAS-trpA1 RNAi*

*EGT; UAS-SERCA RNAi (BL25928)*

**Figure 7—figure supplement 3**

*EGT; UAS-RyR RNAi*

*EGT; UAS-RyR RNAi/ UAS-Ras1^A^*
